# Does Health Insurance Eligibility Improve Child Health: Evidence From the National Health Insurance Scheme (NHIS) in Nigeria

**DOI:** 10.7759/cureus.28660

**Published:** 2022-09-01

**Authors:** Uche E Ekhator-Mobayode, Shailendra Gajanan, Chukwuyem Ekhator

**Affiliations:** 1 Health Economics, The World Bank Group, Washington DC, USA; 2 Micro-Economics, University of Pittsburgh, Bradford, Pittsburgh, USA; 3 Neuro-Oncology, New York Institute of Technology, Old Westbury, USA

**Keywords:** health insurance eligibility, nigeria, difference in difference, child health, national health insurance reform

## Abstract

Favorable child health outcomes are important for sustainable growth and development, especially for developing economies. However, Nigeria has some of the worst health indicators. The problem seems to be inadequate access to affordable healthcare, especially for children. To improve policies aimed at improving access to affordable healthcare for children in Nigeria through health insurance, it is important to measure the extent to which health insurance affects child health. This study examines the effects of health insurance on child health and healthcare utilization in Nigeria using the implementation and expansion of the National Health Insurance Scheme (NHIS) to introduce the exogenous variation in health insurance eligibility, a natural experiment that fits a difference-in-difference model. The findings suggest that health insurance increases birth weight. It also increases the probability that children receive polio and diphtheria vaccines. The findings suggest that the NHIS in Nigeria is effective in improving the health outcomes of children. Policies strengthening the take-up of the NHIS should be encouraged across all sectors and socio-economic groups in the economy.

## Introduction

In developing economies, favorable child health outcomes are important for sustainable growth and development [1]. This is because the long-term effects of childhood health shocks are likely to be larger as it is less likely that these shocks are adequately compensated [2-9]. Early life health shocks can restrict a person’s potential to make a living later in life and consequently affect the development of human capital in a country [10-15]. This presents serious challenges in developing economies because the marginal benefit of human capital is greater than in their developed counterparts [16]. Generally, when there are health shocks, health insurance provides a means of access to healthcare that would otherwise be unaffordable [17-20]. Lack of health insurance exposes individuals to a significant risk of major health expenditures [16-20]. This has led to global discussions of health insurance policies with special concerns for disadvantaged socio-economic groups [14,15]. One important aspect of this debate is the choice of financing health care. The possibilities include a government-funded health insurance scheme, private insurance, or out-of-pocket spending.

Private health insurance in developing economies can be problematic because it depends on a developed financial system that may not be available [14,20]. Also, reliance on out-of-pocket payments results in healthcare services provided based on the ability to pay [21-29], which systematically excludes disadvantaged socioeconomic groups including children [30-36]. The 2005 World Health Assembly called for universal coverage in health systems to help improve global health outcomes [7,22]. However, Nigeria still reports poor child health indicators [16,26]. This situation could be greatly improved by implementing inclusive health insurance policies that reduce the burden of health shocks and increase access to effective and affordable healthcare.

The existing literature generally suggests that health insurance increases health care utilization for children [9,20,24-32,37-41]. Although greater health insurance coverage can improve health outcomes, it is not always the case that increased healthcare because of health insurance translates to more favorable child health outcomes. This may be because healthcare utilization for children is sometimes unnecessary [27]. However, the overuse of healthcare because of health insurance is unlikely to be the case in Nigeria. The problem seems to be inadequate access to affordable healthcare, especially for children [21-24]. To improve policies aimed at improving access to affordable healthcare for children in Nigeria through health insurance, it is important to measure the extent to which health insurance in Nigeria affects child health [25,28,29]

The causality between health insurance coverage and health outcomes is difficult to establish. This is because random variation in health insurance status is not easily observable [31]. Individuals vary by more than just differences in their health insurance coverage [32-35]. In addition, health insurance may affect health status because it may increase healthcare utilization. People may also have health insurance because of pre-existing poor health [19,37-42]. This results in the potential endogeneity of health insurance [43-46,47-50]. One way to deal with the endogeneity problem is to explain the causal effect of health insurance on health outcomes by using an exogenous event to introduce variation in health insurance coverage [31,37-41,50]. This study adds to the growing body of works that use the implementation of health insurance reforms in a country to introduce the exogenous variation in health insurance eligibility [1].

In this study, we quantify the impact of health insurance eligibility under the Nigerian National Health Insurance Scheme (NHIS) on the health status and healthcare utilization of under-five-year-olds. The NHIS was created to improve access to affordable healthcare and better the health status of all Nigerians in compliance with the World Health Organization (WHO) universal health care goal. However, before this study, no empirical evidence on the effect of the NHIS reform on health outcomes using a nationally representative dataset has been reported for Nigeria. We provide evidence on the effect of the NHIS on the prevalence of cough and diarrhea, mortality, birth weight, and the prevalence of vaccination. We use data from the Nigerian Demographic and Health and Survey (NDHS) and employ a difference-in-difference model exploiting the variation in NHIS eligibility across sectors.

The results suggest that the implementation of the NHIS with the Formal Sector Social Health Insurance Program (FSSHIP) increased the probability that children received the complete dosage of vaccines for polio and diphtheria by 9.4% and 10.1% points respectively. The NHIS expansion beyond the formal sector decreased the prevalence of diarrhea by 4.4% points. It also increased the probability that children from middle-income households received medical treatment for diarrhea by about 53% points. These results suggest that the NHIS may be useful to improve child health outcomes in Nigeria. The remainder of the study is organized as follows. The following section provides a background of the NHIS. Section three presents a theoretical framework while Section four describes the data and presents the identification and estimation strategy. Section five presents the estimation results while section six concludes the study.

The National Health Insurance Scheme (NHIS) in Nigeria

Nigeria has some of the worst health indicators in Africa [43-46,51-56]. It has the highest prevalence of malaria globally and accounts for 10% of infant and child mortality even though only 2% of the world’s population resides there. The country is the second-largest contributor to global under-five mortality, with the deaths of about 2,300 under-five-year-olds daily [55-56]. Malaria and diarrhea contribute significantly to the unfavorable under-five health outcomes in Nigeria. Although child healthcare services and delivery are clearly an important issue for the country’s development, the federal government allocated only 4.3% of the 2016 budget to healthcare [43-46]. Healthcare spending in Nigeria ranks below that of other African economies: Nigeria allocates only $67 per capita, while South Africa and Angola allocate seven and three times more respectively.

Health insurance is one way to improve access to affordable healthcare. Prior to successfully signing the National Health Insurance Scheme (NHIS) into law in 1999, there were failed attempts at establishing a health insurance scheme in Nigeria [43]. In 1962, the Minister for Health presented a parliamentary bill for the establishment of a national health insurance scheme. The proposal was revisited in 1988, with another Minister of Health recommending the establishment of a health insurance scheme. However, no law establishing a national health insurance policy was passed until the establishment of the NHIS in 1999. Despite the legislation, the NHIS was not funded in the national budget until 2005. Its implementation began with the Formal Sector Social Health Insurance Program (FSSHIP) in compliance with the World Health Organization (WHO) universal health care goal to improve access to healthcare [1] The FSSHIP provides health insurance coverage for employees in the public sector, organized private sector, armed forces, police, and other uniformed services and their dependents.

Under the FSSHIP, federal government employees and private sector businesses with ten or more employees are required to register. Employees in other tiers of government and small businesses may decide whether to participate. At initial implementation, most participants in the FSSHIP were federal government employees. Since then, the NHIS has designed other programs to ensure universal coverage. The expansion began gradually in 2010 with social health insurance (Dogo-Mohammad, 2010). Presently, there are many programs to suit various socio-economic groups in the country. The other programs are categorized under the Informal Sector Social Health Insurance Programs (ISSHIP) and the Vulnerable Group Social Health Insurance Program (VGSHIP) [3].

Programs under the NHIS are funded differently. With the FSSHIP, contributions are based on earnings: Federal government employees contribute 1.75% of their consolidated earnings while the federal government contributes 3.5%. Employers in the private sector and other levels of government may contribute the entire amount or 10% of the employees’ basic earnings while employees contribute 5%. Participants in other programs pay an annual lump sum contribution or daily, weekly, or monthly contributions. Federal, state, and local government, development partners, and civil society organizations make contributions to the vulnerable group fund. 

The basic benefit is comprehensive. It covers primary healthcare including out-patient care, prescription drugs, pharmaceutical care, and diagnostic tests, maternity care, newborns for twelve weeks from the date of delivery, preventive care (including vaccinations), consultation with specialists, hospital care, eye care, prostheses made in Nigeria, and preventive dental care [1] The exclusions comprise of injuries sustained at work (including extreme sports) and disaster-related injuries (including natural disasters and insurgencies), transplant and cosmetic procedures, drug abuse/addiction treatment, epidemics, family planning commodities, domiciliary treatment, advanced procedures [including complex congenital abnormalities, artificial insemination, and in vitro fertilization (IVF)], and postmortem examination [30]. The contributions made under the FSSHIP benefit the employee, a spouse, and four biological children under 18 years. Employers may also make additional contributions for other benefits and coverage for more children. Contributions under other programs cover the beneficiary for which payment is made. 

Since its initial implementation, the NHIS has made some progress in seeking to protect households from the burden of huge medical bills and ensure access to good healthcare services [1] However, it is not clear how effective the NHIS has been in achieving its objectives. Many Nigerians still rely on out-of-pocket spending to meet their healthcare needs. Although private health insurance seems like an alternative, only about one million Nigerians out of a population of over 170 million have private health insurance [2] Also, the take-up rate of the NHIS has been low. This could be because participation in the scheme is only binding for federal government employees. Private sector employers may opt-out to evade taxes. In 2011 only about five million out of over 150 million Nigerians were enrolled in the NHIS [43-45,51-59]. The next section presents the theoretical framework.

## Materials and methods

Theoretical framework

Health insurance can affect health status by reducing the price of health care. It may also lead to a moral hazard problem [44-45,51-57]. Moral hazards can cause the overconsumption of health care without a change in health behavior, other things equal. The overconsumption of health care that arises may also be because health insurance causes individuals to take greater health risks. However, without loss of generality, it can be argued that moral hazard caused by increasing risky behaviors can be ignored [5]. This is because even though health insurance covers the cost of health care, it does not fully protect against the physical and emotional burden of ill health that may be caused by risky behavior. In other words, it is reasonable to assume that the reduction in the price of health care is the mechanism through which health insurance affects health status.

Based on this assumption, We present a household utility maximization problem subject to a health production function and budget constraint [1] The preference relation is complete, transitive, continuous, strictly monotonic, and strictly convex. The utility can thus be represented by a utility function U that is continuous, strictly increasing and strictly quasiconcave. The problem is as follows:

Max U(C,H) subject to

(2.1) $H = H(B_{m}, X)$ Health production function

(2.2) $P_{c}C + P_{BM}B_{M}\leq Y$ Household budget constraint

where, C = Consumption goods, H = Health status,  B_m_= consumption of health goods or healthcare utilization such as vaccination, X = Socioeconomic and other exogenous factors that affect health, Y = Income,  P_c_= Price of consumption goods, and P_BM_ = Price of health goods. The vector of prices, P>>0, is fixed. Also, the price of consumption goods is normalized to one. The solutions to the problem yield the Marshallian demand functions as follows:

(2.3) $B_{M}^{M}(P_{BM},P_{_{Bn},Y})$

(2.4) $H^{m} = H^{m}(P_{BM},P_{Bn},Y)$

The Slutsky equations showing the effects of the price of healthcare on healthcare utilization, and health status are written below:

(2.5) $\frac{\delta B_{m}^{m}}{\delta P_{m}} = \frac{\delta B_{m}^{h}}{\delta P_{m}} - \frac{\delta B_{m}^{m}}{\delta P_{m}}B_{m}^{m}$

By quasiconcavity of the utility function $\frac{\delta B_{m}^{m}}{\delta P_{m}} \leq 0$. If health goods are normal goods then $\frac{\delta B_{m}^{m}}{\delta Y}> 0$ and it follows that $\frac{\delta B_{m}^{m}}{\delta P_{m}} \leqslant 0$. If they are inferior goods then $\frac{\delta B_{m}^{m}}{\delta Y}&lt; 0$, so that \frac{\delta B_{m}^{m}}{\delta P_{m}} \leq 0\\ if the substitution effect is greater than the income effect.

Hypothesis 2.1

A reduction in the price of healthcare (an increase in health insurance coverage) improves healthcare utilization, other things equal.

(2.6) $\frac{\delta H^{m}}{\delta P_{m}} = \frac{\delta H^{h}}{\delta P_{m}}- \frac{\delta H^{m}}{\delta Y}H^{m}$

Finally, the effect of the price of health goods on health status depends on the pure substitution effect and the income effect. Health status is dependent on healthcare utilization so the total effect is not clear. However, I argue that as income increases, an individual can make better choices and health status improves. Also, cheaper healthcare can directly affect health status by allowing for the utilization of healthcare that would otherwise not be affordable. In other words, $\frac{\delta H^{h}}{\delta P_{m}}&lt; 0$ and $\frac{\delta H^{m}}{\delta Y}> 0$ so that $\frac{\delta H^{m}}{\delta P_{m}}&lt; 0.$.

Hypothesis 2.2

A reduction in the price of healthcare (an increase in health insurance coverage) improves health status, other things equal.

Data, identification, and estimation strategy

The Nigerian Demographic and Health Surveys (NDHS)

The study sample is from the Children’s Recode (KR) of 2003, 2008, and 2013 Nigerian Demographic and Health Survey (NDHS). The NDHS is a nationally representative cross-sectional household survey. Women between 15 and 49 years of age in the surveyed households are interviewed individually. The KR has one record for every child between 0 and 59 months of age born to these women including children who are dead. It contains information on the child’s birth weight, vaccinations, the occurrence of infectious diseases, and whether the child received treatment for these diseases. It also contains demographic information including age or age at death, education level, occupation, and employment status of a child’s mother and her husband/partner [1] The KR for 2003, 2008, and 2013 NDHS contains 6029, 28,647, and 31,482 observations, respectively.

Outcome Variables

Child health indicators: The effect of the NHIS on the health status of children under five is estimated using the prevalence of cough and diarrhea as proxies. We also estimate the effect of the NHIS on child mortality and birth weight. These variables derive from questions answered by the mother in the NDHS. The mother is asked if the child had a cough or diarrhea in the two weeks preceding the survey for the prevalence of cough and diarrhea. The variable showing the prevalence of cough (diarrhea) is a binary variable equal to one if the child had a cough (or diarrhea) and 0 otherwise. The mortality variable is also an indicator variable equal to one if the child is dead and 0 otherwise. Finally, birth weight is measured in kilograms and is derived by asking the mother about the child’s birth weight. She reports the child’s birth weight by recalling it from memory or showing the interviewer the child’s birth card. For accuracy, We restrict the observations used in the analysis of birthweight to those reported on the child’s birth card. The mother’s perception of illness is not validated by a medical examiner during the survey. The reports given by her about the child’s health are, however, good proxies for the child's health status [44].

Healthcare utilization indicators: The effect of the NHIS on healthcare use of children under age five is estimated using the prevalence of medical treatment for cough/malaria, the prevalence of medical treatment for diarrhea, and the prevalence of vaccination (polio0, polio1, polio2, polio3, Bacillus Calmette-Guérin (BCG), measles, diphtheria1, diphtheria, and diphtheria3). These variables derive from questions answered by the mother. For the prevalence of medical treatment of cough or malaria, the mother is asked if the child had medical treatment for cough or fever if the child had cough or fever. The variable that shows the prevalence of medical treatment of cough or malaria is a binary variable equal to one if the child had medical treatment for cough or fever given that the child had cough or fever and 0 otherwise. For the prevalence of medical treatment for diarrhea, the mother is asked if the child had medical treatment for diarrhea if the child had diarrhea. The variable that shows the prevalence of medical treatment for diarrhea is a binary variable equal to one if the child had medical treatment for diarrhea given that the child had diarrhea and 0 otherwise. Finally, for the child’s vaccinations, the mother is asked if the child was vaccinated. The mother reports the child’s vaccination history either by recalling it from memory or by presenting the child’s vaccination card to the interviewer. For accuracy, We restrict my analysis to observations of children where the interviewer saw the child’s vaccination card as proof of the child’s vaccination. The variable that shows the prevalence of a vaccination is a binary variable equal to one if the child had the vaccination and 0 otherwise.

Identification and Estimation Strategy

Identification: To identify the effect of the NHIS on child health outcomes, observations of children from the 2003 NDHS provide data for the period before the initial implementation of the NHIS with the FSSHIP. Observations of children from the 2008 sample provide data for the period after the initial implementation, but before the expansion of the NHIS with other programs. Finally, observations of children from the 2013 NDHS provide data for the period after the implementation and expansion of the NHIS. The first step in identifying the effect of the NHIS is to assign eligibility to each child. The NDHS does not explicitly report the sector in which a child’s parents are employed. We use the information provided about the occupation of a child’s parents to determine eligibility for health insurance coverage [1] A child with at least one parent in the formal sector is eligible for coverage at the initial implementation of the NHIS under the FSSHIP. However, this identification is potentially endogenous if people self-select into the formal sector so that they are eligible for health insurance coverage under the NHIS. This may also be likely even after NHIS eligibility opened to other sectors because employees in the formal sector pay only a portion of the required premium and are automatically eligible through their employer.

To deal with this potential endogeneity, We restrict the formal sector in the study sample to public sector employees including armed forces, police, and other uniformed services officers (referred to as the public sector hereafter). This restriction addresses the issue reasonably because while it may be relatively easy to switch to the private sector depending on the state of the economy, the reality in the public sector is not the same. Public sector job creation in Nigeria is declining or minimal at best due to privatization [10]. This results from the theft of public funds and/or politicians’ lack of willingness to expand the bureaucracy in the public sector. These jobs are therefore relatively fixed with high job tenure rates and relatively low risk of layoff. The implications of this are little or no job creation in the public sector and a high barrier to entry. Therefore, it is unlikely that people could self-select into the public sector to be eligible for health insurance coverage under the NHIS after its implementation.

To estimate the effect of the implementation of the NHIS, children with at least one parent in the public sector are the treatment group since they were eligible at the initial implementation phase. Those with both parents in other sectors are the control group because they became eligible after expansion beyond the formal sector began. To identify the effect of the expansion of the NHIS, children with both parents in other sectors are the treatment group since they became eligible after expansion, while those with at least one parent in the public sector are the control group because of their eligibility for coverage under the NHIS was not affected by the expansion of the NHIS. Other sectors are defined as the unemployed, merchants, traders, salesmen, shop assistants, and other related occupations (referred to as other sectors hereafter) [1]. A further argument in favor of restricting the formal sector to only public-sector employees is the fact that the NHIS Act requires only federal government employees to be registered. Employees in the organized private sector may opt out of the scheme. A full list of occupations in the public sector and other sectors for this study is in Appendix A.

Estimation strategy: We estimate the following difference-in-difference (DID) model to examine the effect of the NHIS on child health outcomes:

(2.7) $H_{ist} = \beta _{0} +\beta _{1}PostImp_{ist} + \beta _{2}PostExp_{ist} + \beta _{3}Public_{is} + \beta _{4}PostImp_{ist}$ * $Public_{is} + \beta _{5}PostExp_{ist}$ * $Public_{is} + \beta _{6}X_{ist} +\delta ^{!}NDHSYear + \varphi ^{!}Month + Y^{!}State + \varepsilon _{ist}$

The outcome variable is a measure of health or healthcare utilization for child ‘i’ with a parent in sector ‘s’ and mother interviewed in NDHS year ‘t’. Indicates whether the child’s observation is after the initial implementation but before the expansion of the NHIS; it is one for observations of children from the 2008 and 2013 NDHS and 0 otherwise (in the birth weight equation, it is one for children born after 2005 and 0 otherwise). Indicates whether the child’s observation is before or after the expansion of the NHIS; it is one for observations of children from the 2013 NDHS and 0 otherwise (in the birth weight equation, it is one for children born after 2010 and 0 otherwise). Is the variable allocating treatment. It indicates whether the child has at least one parent in the public sector. It is one for observations of children with at least one parent in the public sector and 0 otherwise. Is the set of control variables. All equations control for the demographic characteristics of the child, parents, and household. These characteristics include the child’s gender, mother’s age, mother’s age square, mother’s education, father’s age, father’s education, birth order, household size, religion, and whether the family lives in an urban or rural area. Because extra payments are made to cover more than four children, the explanatory variables also include family income, although family wealth is used as a proxy for income. The wealth indicator is a reasonable proxy for income. The NDHS Report states, “the wealth index is consistent with income and expenditure measures. The index is calculated using household asset data via a principal component analysis. It considers urban-rural differences and indicators of wealth” (NDHS, 2013).

We also control for drought [1]. Drought can add to water and food shortages leading to famine and it may affect child health outcomes [45]. We also include controls for child’s age and child’s age square except in the mortality and birth weight equations. In addition, the prevalence of diarrhea and the prevalence of medical treatment of diarrhea equations control for water source and type of toilet in the household.  accounts for the round of the NDHS survey. Accounts for the month of the interview, capturing seasonality across months that may affect health outcomes (month of interview fixed effects are excluded from the birthweight equation. We instead include year and month of birth to capture unobserved characteristics across years and months that may affect birth weight). Accounts for state fixed effects. Nigeria is divided into states and administrative differences in states may affect how the NHIS impacts health outcomes. Is the child-specific error. We cluster the standard errors at the NDHS survey cluster level. The clusters represent the sample points used during the NDHS fieldwork. The full sample has 904 clusters. Finally, we account for the stratification used in the NDHS sampling design.

The DID estimator for the NHIS implementation in the public sector is $\beta _{4}$ [12][see Appendix B]. The DID estimator for the expansion of the NHIS to other sectors is -$\beta _{5}$ [13][see Appendix B]. Equation (2.7) is estimated with Ordinary Least Squares (OLS). Since the goal of the NHIS is to improve the health status of all Nigerians, we expect that the average treatment effect of the implementation and expansion of the NHIS on the health outcomes of children is favorable. Specifically, we expect that the implementation and expansion of the NHIS increase birth weight, the prevalence of vaccinations, the prevalence of medical treatment for diarrhea, and the prevalence of medical treatment for cough or malaria. we also expect that the implementation and expansion of the NHIS reduces the prevalence of cough, diarrhea, and child mortality. The next section presents the estimation results.

## Results

Description of the study sample

Table 1 presents a description of the study sample used in the analysis.

**Table 1:** Description and Summary Statistics of Variables Used in the Analysis The numbers reported are the mean of each variable. Standard deviations are in parenthesis. BCG: Bacillus Calmette-Guérin, DPT: diphtheria-pertussis-tetanus

		Public Sector		Other Sectors		Total
Variables	Description	Before NHIS	After NHIS	Before NHIS	After NHIS	
DEPENDENT VARIABLES						
a. Health Indicators						
Cough	=1 if child had cough in the two weeks preceding the survey =0 otherwise	0.254 (0.436)	0.163 (0.369)	0.214 (.411)	0.155 (0.362)	0.168 (0.373)
Diarrhea	=1 if child had diarrhea in the two weeks preceding the survey =0 otherwise	0.143 (0.350)	0.073 (0.261)	0.223 (0.416)	0.114 (0.317)	0.115 (0.319)
Mortality	=1 if child died =0 otherwise	0.110 (0.313)	0.067 (0.250)	0.119 (0.324)	0.101 (0.302)	0.096 (0.295)
Birthweight	Weight at birth in kilograms	3.308 (0.645)	3.324 (0.719)	2.974 (0.869)	3.334 (0.740)	3.312 (0.737)
b. Healthcare Utilization						
Medical Treatment for Cough/Malaria	=1 if had medical treatment for cough or fever given that child had cough or fever =0 otherwise	0.394 (0.491)	0.526 (0.500)	0.321 (0.468)	0.419 (0.494)	0.422 (0.494)
Medical Treatment for Diarrhea	=1 if had medical treatment for diarrhea given that child had diarrhea =0 otherwise	0.370 (0.488)	0.486 (0.502)	0.206 (0.406)	0.353 (0.478)	0.349 (0.477)
Polio0	=1 if had Polio0 vaccine =0 otherwise	0.228 (0.420)	0.521 (0.500)	0.130 (0.336)	0.217 (0.412)	0.264 (0.441)
Polio1	=1 if had Polio1 vaccine =0 otherwise	0.433 (0.497)	0.684 (0.465)	0.233 (0.423)	0.348 (0.476)	0.414 (0.493)
Polio2	=1 if had Polio2 vaccine =0 otherwise	0.329 (0.471)	0.594 (0.491)	0.162 (0.369)	0.270 (0.443)	0.329 (0.470)
Polio3	=1 if had Polio3 vaccine =0 otherwise	0.160 (0.368)	0.407 (0.492)	0.094 (0.293)	0.187 (0.390)	0.225 (0.418)
BCG	=1 if had BCG vaccine =0 otherwise	0.459 (0.500)	0.647 (0.478)	0.201 (0.401)	0.256 (0.437)	0.326 (0.469)
DPT1	=1 if had DPT1 vaccine =0 otherwise	0.314 (0.465)	0.600 (0.490)	0.160 (0.367)	0.237 (0.425)	0.297 (0.457)
DPT2	=1 if had DPT2 vaccine =0 otherwise	0.242 (0.429)	0.518 (0.500)	0.120 (0.325)	0.196 (0.397)	0.248 (0.432)
DPT3	=1 if had DPT3 vaccine =0 otherwise	0.180 (0.385)	0.436 (0.496)	0.088 (0.283)	0.161 (0.367)	0.206 (0.404)

CONTROL VARIABLES						
a. Child						
Age	Age in months	27.301 (16.702)	28.522 (16.924)	25.910 (17.173)	27.455 (17.251)	27.546 (17.169)
Age^2^	Age in months squared	1023.383 (981.604)	1099.742 (1017.334)	965.863 (996.661)	1051.317 (1022.109)	1053.51 (1018.137)
Male	Child is male	0.521 (0.500)	0.512 (0.500)	0.520 (0.500)	0.501 (0.500)	0.506 (0.500)
b. Parents						
Mother’s age	Mother’s age in years	31.263 (6.694)	30.930 (5.915)	27.889 (6.855)	28.804 (6.976)	29.239 (6.828)
Mother’s age square	Mother’s age in years squared	1022.058 (426.675)	991.632 (377.742)	824.734 (413.299)	878.331 (432.009)	901.512 (423.409)
Mother’s education	Mother's education in single years	6.912 (5.279)	9.501 (5.332)	3.580 (4.694)	4.171 (5.164)	5.285 (5.605)
Father’s age	Father’s age in years	42.897 (9.067)	40.75 (7.877)	38.705 (9.617)	39.789 (9.566)	39.994 (9.274)
Father’s education	Father’s education in single years	10.688 (5.047)	12.991 (3.726)	5.080 (5.268)	5.529 (5.623)	7.147 (6.076)
c. Household						
Birth order	Order born in the family	4.359 (2.609)	3.590 (2.319)	3.828 (2.580)	3.946 (2.634)	3.879 (2.574)
Household size	Number of household members	7.110 (0.039)	6.928 (3.847)	7.315 (3.908)	7.255 (3.788)	7.189 (3.789)
Urban	=1 if residence in urban area =0 otherwise	0.537 (0.499)	0.617 (0.486)	0.428 (0.495)	0.377 (0.485)	0.435 (0.496)
Christian	=1 if mother is Christian (omitted) =0 otherwise	0.496 (0.498)	0.566 (0.570)	0.245 (0.089)	0.276 (0.095)	0.339 (0.185)
Islam	=1 if mother is Muslim =0 otherwise	0.504 (0.501)	0.428 (0.494)	0.752 (0.432)	0.719 (0.449)	0.656 (0.475)
Other religion	=1 if mother practices other religion =0 otherwise	0.000 (0.000)	0.006 (0.077)	0.003 (0.058)	0.005 (0.074)	0.005 (0.072)
Wealth (lowest quintile)	=1 if household is in lowest quintile of the NDHS wealth index (omitted) =0 otherwise	0.066 (0.086)	0.020 (0.130)	0.130 (0.205)	0.207 (0.395)	0.159 (0.350)
Wealth (second quintile)	=1 if household is in second quintile of the NDHS wealth index (omitted) =0 otherwise	0.132 (0.338)	0.056 (.231)	0.196 (0.397)	0.241 (0.427)	0.196 (0.397)
Wealth (middle quintile)	=1 if household is in middle quintile of the NDHS wealth index (omitted) =0 otherwise	0.140 (0.347)	0.158 (0.365)	0.231 (0.422)	0.183 (0.387)	0.181 (0.385)
Wealth (fourth quintile)	=1 if household is in fourth quintile of the NDHS wealth index (omitted) =0 otherwise	0.282 (0.451)	0.280 (0.449)	0.259 (0.438)	0.188 (0.391)	0.215 (0.411)
Wealth (highest quintile)	=1 if household is in highest quintile of the NDHS wealth index (omitted) =0 otherwise	0.380 (0.486)	0.486 (0.500)	0.184 (0.388)	0.181 (0.385)	0.249 (0.433)
Drought	=1 if residence in a state affected by drought	0.789 (0.409)	0.770 (0.421)	0.849 (0.359)	0.723 (0.448)	0.745 (0.436)
Toilet	=1 if household’s type of toilet facility is flush toilet =0 otherwise	0.282 (0.450)	0.415 (0.493)	0.118 (0.322)	0.153 (0.360)	0.207 (0.405)
Water Source	=1 if source of drinking water is protected/piped water =0 otherwise	0.597 (0.491)	0.837 (0.369)	0.432 (0.496)	0.619 (0.486)	0.646 (0.478)
Number of Observations		365 (3.38%)	2,167 (20.08%)	892 (8.27%)	7,368 (68.27%)	10,792 (100%)

There are 10,792 observations in the full sample. Twenty-three percent of children in the sample have at least one parent in the public sector while about 77% have both parents in other sectors. About 12% of the children in the sample provide data for the period before the NHIS implementation while 88% provide data for the period after.

Children with at least one parent in the public sector have a higher prevalence of cough but a lesser prevalence of diarrhea before and after the NHIS implementation compared to those with both parents in other sectors. The proportion of children who had a cough in the public sector before the NHIS is 25.4%, compared to 21.4% in other sectors and 16.8% for the full sample. About 16.3% of children in the public sector after the NHIS had cough compared to 15.5% in other sectors. The proportion of children in the public sector who had diarrhea before the NHIS is 14.3% compared to 22.3% in other sectors and over 11.5% for the full sample. Also, 7.3% of children in the public sector after the NHIS had diarrhea compared to 11.4% in other sectors. Child mortality was higher before the NHIS than after. The proportion of children who are dead before the NHIS is 11% for the public sector and 11.9% for other sectors, compared to 6.7% and 10.1% after the implementation of the NHIS. Finally, children in the public sector weigh more at birth on average. They have an average birthweight of 3.31kg compared to 2.97kg for children in other sectors. The reverse is the case after the NHIS when children in other sectors have a slightly higher average birthweight of 3.33kg compared to 3.32kg for those in the public sector.

For healthcare utilization, children in the public sector have a higher prevalence of medical treatment for cough, malaria, and diarrhea than those in other sectors before and after the NHIS. 39.4% and 52.6% of children had medical treatment for cough or fever in the public sector before and after the NHIS respectively. In other sectors, 32.1% and 41.9% had medical treatment for cough or fever before and after the NHIS respectively. 37% and 48.6% of children in the public sector had medical treatment for diarrhea before and after the NHIS respectively, while 20.6% and 35.3% of those in other sectors had medical treatment for diarrhea before and after the NHIS respectively. Finally, the prevalence of polio0, polio1, polio2, polio3, BCG, diphtheria-pertussis-tetanus (DPT)1, DPT2, and DPT3 vaccination are all higher for children in the public sector compared to those in other sectors both before and after the NHIS.

Children in the public sector are slightly older on average. The average age of children in the public sector before the NHIS is 27 months compared to an average age of 28.52 months for children after the NHIS. For children in other sectors, the corresponding numbers are 25.91 and 27.46 months respectively. The proportion of boys and girls in the sample is almost even both before and after the NHIS. The average age of the mothers and fathers are 29.23 and 39.99 years respectively for the full sample. Parents of children in the public sector seem to be older. The average age of mothers of children in the public sector before and after the NHIS is 31.26 and 30.93 years respectively compared to 27.89 and 28.80 for those in other sectors. The average age of fathers of children in the public sector before and after the NHIS is 42.90 and 40.75 years respectively compared to 38.71 and 39.79 for those in other sectors.

More education tends to correlate with waiting longer to have children. It is, therefore, no surprise that parents of children in the public sector are better educated than parents of children in other sectors. Mothers of children in the public sector have an average of 6.91 and 9.50 years of education before and after the NHIS respectively, compared to an average of 3.58 and 4.17 years of education for mothers in other sectors before and after the NHIS respectively. Fathers of children in the public sector have an average of 10.69 and 12.99 years of education before and after the NHIS respectively, compared to an average of 5.08 and 5.53 years of education for fathers in other sectors before and after the NHIS respectively.

Finally, a larger proportion of children in the public sector are from wealthier households. The proportion of children from households in the lowest wealth quintile in the public sector before and after the NHIS is 6.6% and 2% respectively compared to 13% and 20.7% in other sectors. The proportion of children from households in the highest wealth quintile in the public sector before and after the NHIS is 38% and 48.6% respectively, compared to 18.4% and 18.1% in other sectors. In summary, the description of the sample does not clearly show whether child health outcomes were more favorable in the public sector than in other sectors before the NHIS. Although children in the public sector had a lesser prevalence of diarrhea, higher average birthweight, and lesser mortality before the NHIS compared to children in other sectors, they also had a higher prevalence of cough. However, children in the public sector had higher healthcare utilization than those in other sectors, which seems reasonable since they have older and more educated parents and are more likely to be from wealthier households. In the next section, we present the estimation results.

Estimation Results

All models are estimated for the full sample as well as sub-samples grouped by household’s level of income as follows: low-income, middle-income, and high-income [1].

Health Status

The estimates of the average treatment effect of the NHIS implementation and expansion on child health status are reported in Table 2.

**Table 2:** The Impact of the NHIS on Health Status The standard errors in parentheses are robust and adjusted for at least 120 clusters in each equation. An estimate of 0.000 for both the coefficient and standard errors indicates too few observations for the estimation. ***Statistically significant at 0.01 level. **Statistically significant at 0.05 level. *Statistically significant at 0.10 level.

DEPENDENT VARIABLE	IMPLEMENTATION	EXPANSION
PANEL A: FULL SAMPLE		
Prevalence of Cough	0.009 (0.090)	0.017 (0.086)
Prevalence of Diarrhea	0.043 (0.030)	-0.044** (0.025)
Mortality	0.004 (0.027)	0.005 (0.020)
Birthweight (kg)	0.159 (0.161)	0.269* (0.138)
PANEL B: LOW-INCOME SAMPLE		
Prevalence of Cough	-0.244 (0.267)	-0.192 (0.226)
Prevalence of Diarrhea	0.141 (0.137)	-0.108 (0.099)
Mortality	-0.038 (0.080)	0.099 (0.067)
Birthweight (kg)	0.000 (0.000)	0.000 (0.000)
PANEL C: MIDDLE-INCOME SAMPLE		
Prevalence of Cough	-0.231 (0.190)	-0.206 (0.132)
Prevalence of Diarrhea	-0.053 (0.106)	-0.011 (0.058)
Mortality	0.092 (0.089)	0.047 (0.053)
Birthweight (kg)	0.000 (0.000)	0.000 (0.000)
PANEL D: HIGH-INCOME SAMPLE		
Prevalence of Cough	-0.046 (0.087)	-0.014 (0.078)
Prevalence of Diarrhea	0.052 (0.036)	-0.009 (0.021)
Mortality	-0.010 (0.031)	-0.015 (0.023)
Birthweight (kg)	0.174 (0.170)	0.280** (0.142)
State Fixed Effects	Yes	Yes
Month of Interview Fixed Effects (Month of birth for birthweight estimation)	Yes	Yes

Estimates for the full sample suggest that the NHIS had no significant effect on the prevalence of cough and mortality. The NHIS expansion only had significant effects on the prevalence of diarrhea and mortality. It reduced the prevalence of diarrhea by 4.4% points and increased birthweight by 0.269kg. Estimates for the sub-samples by household income level also suggest that the NHIS had no significant effect on the prevalence of cough, the prevalence of diarrhea, or mortality. The NHIS expansion also increased the birth weight of children from high-income households by 0.280 kilograms.

Healthcare Utilization

Estimates for the full sample suggest that the NHIS had no significant effect on the prevalence of cough and mortality. The NHIS expansion only had significant effects on the prevalence of diarrhea and mortality. It reduced the prevalence of diarrhea by 4.4% points and increased birthweight by 0.269kg. Estimates for the sub-samples by household income level also suggest that the NHIS had no significant effect on the prevalence of cough, the prevalence of diarrhea, or mortality. The NHIS expansion also increased the birth weight of children from high-income households by 0.280 kilograms.

Medical Treatment

The estimates of the average treatment effect of the NHIS implementation and expansion on the prevalence of medical treatment are reported in Table 3.

**Table 3:** The Impact of the NHIS on Medical Treatment The standard errors in parentheses are robust and adjusted for at least 120 clusters in each equation. **Statistically significant at 0.05 level. *Statistically significant at 0.10 level.

DEPENDENT VARIABLE	IMPLEMENTATION	EXPANSION
PANEL A: FULL SAMPLE		
Prevalence of Medical Treatment for Cough/Malaria	0.056 (0.083)	-0.022 (0.117)
Prevalence of Medical Treatment of Diarrhea	-0.007 (0.157)	0.045 (0.148)
PANEL B: LOW-INCOME SAMPLE		
Prevalence of Medical Treatment for Cough/Malaria	0.238 (0.205)	0.307 (0.315)
Prevalence of Diarrhea Incidence of Medical Treatment of Diarrhea	0.068 (0.229)	0.123 (0.206)
PANEL C: MIDDLE-INCOME SAMPLE		
Prevalence of Medical Treatment for Cough/Malaria	0.298 (0.167)	-0.099 (0.204)
Prevalence of Medical Treatment of Diarrhea	0.473* (0.285)	0.528** (0.243)
PANEL D: HIGH-INCOME SAMPLE		
Prevalence of Medical Treatment for Cough/Malaria	-0.055 (0.106)	-0.152 (0.137)
Prevalence of Medical Treatment of Diarrhea	0.241 (0.238)	-0.052 (0.238)
State Fixed Effects	Yes	Yes
Month of Interview Fixed Effects	Yes	Yes

Estimates for the full sample suggest that the NHIS implementation and expansion had no significant effect on the prevalence of medical treatment for cough or malaria and medical treatment for diarrhea. However, estimates for the sub-samples by household income level suggest that the NHIS implementation and expansion increased the probability that a child from a middle-income household received medical treatment for diarrhea by 47.3% and 52.8% points respectively.

Polio Vaccination

The estimates of the average treatment effect of the NHIS implementation and expansion on the prevalence of polio vaccinations are reported in Table 4.

**Table 4:** The Impact of the NHIS on Polio Vaccination The standard errors in parentheses are robust and adjusted for at least 300 clusters in each equation. **Statistically significant at 0.05 level. *Statistically significant at 0.10 level.

DEPENDENT VARIABLE	IMPLEMENTATION	EXPANSION
PANEL A: FULL SAMPLE		
Prevalence of polio0 vaccination	0.113* (0.072)	-0.021 (0.041)
Prevalence of polio1 vaccination	0.126** (0.054)	0.031 (0.041)
Prevalence of polio2 vaccination	0.081 (0.058)	0.014 (0.038)
Prevalence of polio3 vaccination	0.094** (0.050)	0.013 (0.038)
PANEL B: LOW-INCOME SAMPLE		
Prevalence of polio0 vaccination	0.050 (0.103)	-0.024 (0.097)
Prevalence of polio1 vaccination	-0.079 (0.159)	0.093 (0.173)
Prevalence of polio2 vaccination	-0.041 (0.151)	-0.042 (0.152)
Prevalence of polio3 vaccination	0.035 (0.115)	0.059 (0.147)
PANEL C: MIDDLE-INCOME SAMPLE		
Prevalence of polio0 vaccination	0.075 (0.106)	-0.037 (0.087)
Prevalence of polio1 vaccination	0.075 (0.153)	0.114 (0.115)
Prevalence of polio2 vaccination	-0.099 (0.117)	-0.140 (0.108)
Prevalence of polio3 vaccination	-0.106 (0.119)	-0.079 (0.078)
PANEL D: HIGH-INCOME SAMPLE		
Prevalence of polio0 vaccination	0.106 (0.091)	0.039 (0.050)
Prevalence of polio1 vaccination	0.138* (0.072)	0.094** (0.042)
Prevalence of polio2 vaccination	0.127 (0.075)	0.073 (0.046)
Prevalence of polio3 vaccination	0.118* (0.064)	0.058 (0.052)
State Fixed Effects	Yes	Yes
Month of Interview Fixed Effects	Yes	Yes

Estimates for the full sample suggest that the NHIS implementation and expansion had no significant effect on the prevalence of polio2 vaccination. The NHIS implementation increased the probability that a child would have the polio1 vaccine by 12.6% points and increased the probability that a child would complete the polio vaccine dosage (polio3) by 9.4% points.

Estimates for the sub-samples by household income level suggest that the NHIS implementation and expansion only had a significant effect on polio vaccination for children from high-income households. The NHIS implementation and expansion increased the probability that a child from a high-income household would receive polio1 vaccine by 13.8% and 9.4% points respectively. The NHIS implementation also increased the probability that a child from a high-income household would complete the polio vaccine dosage by 11.8% points.

BCG and Measles Vaccination

The estimates of the average treatment effect of the NHIS implementation and expansion on the prevalence of BCG and measles vaccinations are reported in Table 5.

**Table 5:** The Impact of the NHIS on BCG and Measles Vaccination The standard errors in parentheses are robust and adjusted for at least 400 clusters in each equation. *Statistically significant at 0.10 level. BCG: Bacillus Calmette-Guérin

DEPENDENT VARIABLE	IMPLEMENTATION	EXPANSION
PANEL A: FULL SAMPLE		
Prevalence of BCG vaccination	0.034 (0.051)	0.041 (0.037)
Prevalence of measles vaccination	0.039 (0.039)	0.024 (0.031)
PANEL B: LOW-INCOME SAMPLE		
Prevalence of BCG vaccination	0.050 (0.104)	-0.021 (0.075)
Prevalence of measles vaccination	0.041 (0.098)	-0.009 (0.104)
PANEL C: MIDDLE-INCOME SAMPLE		
Prevalence of BCG vaccination	-0.034 (0.146)	-0.016 (0.084)
Prevalence of measles vaccination	-0.014 (0.094)	-0.090 (0.081)
PANEL D: HIGH-INCOME SAMPLE		
Prevalence of BCG vaccination	0.128 (0.065)	0.079* (0.044)
Prevalence of measles vaccination	0.017 (0.063)	0.074* (0.037)
State Fixed Effects	Yes	Yes
Month of Interview Fixed Effects	Yes	Yes

Estimates for the full sample suggest that the NHIS implementation and expansion had no significant effect on the prevalence of BCG and measles vaccination. Estimates for the sub-samples by household income level suggest that the NHIS expansion increased the probability that a child from a high-income household would receive BCG and measles vaccines by 7.9% and 7.4% points respectively.

DPT Vaccination

The estimates of the average treatment effect of the NHIS implementation and expansion on the prevalence of DPT vaccinations are reported in Table 6.

**Table 6:** The Impact of the NHIS on DPT Vaccination The standard errors in parentheses are robust and adjusted for at least 300 clusters in each equation. **Statistically significant at 0.05 level. *Statistically significant at 0.10 level. DPT: diphtheria-pertussis-tetanus

DEPENDENT VARIABLE	IMPLEMENTATION	EXPANSION
PANEL A: FULL SAMPLE		
Prevalence of DPT1 vaccination	0.131** (0.065)	0.020 (0.041)
Prevalence of DPT2 vaccination	0.117** (0.048)	0.013 (0.040)
Prevalence of DPT3 vaccination	0.101** (0.050)	-0.018 (0.031)
PANEL B: LOW-INCOME SAMPLE		
Prevalence of DPT1 vaccination	0.101 (0.111)	0.039 (0.094)
Prevalence of DPT2 vaccination	-0.004 (0.098)	-0.073 (0.096)
Prevalence of DPT3 vaccination	0.043 (0.082)	-0.041 (0.097)
PANEL C: MIDDLE-INCOME SAMPLE		
Prevalence of DPT1 vaccination	0.010 (0.119)	-0.108 (0.086)
Prevalence of DPT2 vaccination	-0.070 (0.123)	-0.066 (0.087)
Prevalence of DPT3 vaccination	-0.048 (0.107)	-0.066 (0.075)
PANEL D: HIGH-INCOME SAMPLE		
Prevalence of DPT1 vaccination	0.127 (0.084)	0.071 (0.046)
Prevalence of DPT2 vaccination	0.139** (0.057)	0.080* (0.045)
Prevalence of DPT3 vaccination	0.102 (0.063)	0.038 (0.034)
State Fixed Effects	Yes	Yes
Month of Interview Fixed Effects	Yes	Yes

Estimates for the full sample suggest that the NHIS implementation increased the probability that a child had the DPT1, DPT2, and DPT3 vaccines by 13.1%, 11.7%, and 10.1% points respectively. Estimates for the sub-samples by household income level suggest that the NHIS implementation and expansion increased the probability that a child from a high-income household had the DPT2 vaccine by 13.9% and 8% points respectively.

Estimation concerns

A potential issue with the analysis in the previous section is that DID models assume parallel trends in outcomes of the treatment and control groups prior to the NHIS implementation. However, child health outcomes may be more favorable for children with at least one parent in the public sector because of reasons other than the NHIS. This may be due to a differential trend in health outcomes already existing across sectors. The description of the study sample suggested that this trend may exist because children in the public sector had better-educated parents and are more likely to come from wealthier households. To address this concern, we examine children who were not affected by the NHIS. If there were pre-existing trends, children with at least one parent in the public sector should have more favorable health outcomes than children with both parents in other sectors in the cohort not affected by the NHIS. 

Because of a lack of adequate NDHS data before the implementation of the NHIS in 2005 [1], we focus only on birth weight and estimate equation (2.7) using placebo treatments. we compare the birth weight of children born before 2005 (the unaffected cohort) with at least one parent in the public sector to those with both parents in other sectors. These children were not affected by the NHIS. we assume that the unaffected cohort born before 2000 are prior to the implementation of the NHIS, while those born after 2000 are after its implementation but before the expansion, and children born between 2003 and 2005 are after the expansion of the NHIS [2] If child health outcomes were more favorable for children with at least one parent in the public sector, we should find spurious significant coefficients for birthweight; otherwise, the coefficients should be insignificant [3]. The results from this estimation are presented in Table 7. None of the coefficients are statistically significant. We find no evidence that health outcomes were more favorable for children of parents in the public sector before the implementation of the NHIS.

**Table 7:** Evidence in Favor of the Identification Assumption (Birthweight Estimation) The standard errors in parentheses are robust and adjusted for at least 300 clusters in each equation. An estimate of 0.000 for both the coefficient and standard errors indicate too few observations for the estimation.

DEPENDENT VARIABLE	IMPLEMENTATION	EXPANSION
PANEL A: FULL SAMPLE		
Birthweight (kg)	0.027 (0.575)	-0.336 (0.469)
PANEL B: LOW-INCOME SAMPLE		
Birthweight (kg)	0.000 (0.000)	0.000 (0.000)
PANEL C: MIDDLE-INCOME SAMPLE		
Birthweight (kg)	0.000 (0.000)	0.000 (0.000)
PANEL D: HIGH-INCOME SAMPLE		
Birthweight (kg)	-0.078 (0.660)	-0.338 (0.483)
State Fixed Effects	Yes	Yes
Month of Birth Fixed Effects	Yes	Yes

Another issue that may bias the results from estimating equation (2.7) is the potential endogeneity of public sector employment already discussed in section 2.4. Switching to the public sector because of the NHIS is unlikely because public sector job creation in Nigeria ranges from minimal to none because of the corruption and negligence of the political class [10, 51-54].

## Discussion

Preventable and curable diseases have caused a large proportion of the morbidity and mortality of under-five-year-olds in Nigeria. Access to affordable healthcare for Nigerian children is a development issue. The National Health Insurance Scheme (NHIS) was created to increase access to good healthcare services, help households reduce risk from health shocks, and ensure high standards of healthcare delivery. It was initially implemented with the formal sector program in 2005. Expansion to other sectors of the economy began in 2010. This essay uses a difference-in-difference approach to estimate the effect of the implementation and expansion of the NHIS on the health outcomes of under-five-year-olds in Nigeria.

The findings from the essay suggest that the NHIS expansion reduced the prevalence of diarrhea in children under age five by 4.4% points and increased birth weight by 0.269kg. For children from high-income households, the NHIS expansion increased birthweight by 0.28kg. The results also suggest that the NHIS implementation and expansion increased the probability that a child from a middle-income household would receive medical treatment for diarrhea by 47.3% and 52.8% points respectively. Finally, there is evidence that the NHIS implementation increased the probability that a child would be vaccinated for polio and diphtheria. The findings suggest that higher health care utilization to treat diarrhea in children from middle-income households in Nigeria may translate to better health.

These results contravene Kemper’s (1998) suggestion that health utilization by children does not necessarily lead to better health for children in the United States. Kemper’s study reported a substantial rate of inappropriate medical care use for children. This is unlikely to be a problem in a developing country such as Nigeria. The concerns in developing countries are mostly reducing barriers to access to necessary healthcare and reducing costs at the point of care rather than overuse. My findings are also like the results from Mensah et al. (2010) [37], who report a higher utilization rate for pregnancy care and lower levels of infant death among the insured in Ghana. The results do not reflect the effect of the NHIS on the intensive margin because of a lack of adequate data. As additional data become available in the future, it will be interesting to estimate the effect of the timing of take-up of health insurance coverage on the health outcomes of children in Nigeria.

## Conclusions

Finally, although the study sheds light on the effect of the NHIS on child health outcomes, it raises an important question: Why does the NHIS have differential impacts across children from various income groups? There are two possible explanations. First, households’ initial wealth endowment differs across these groups. Healthcare, although very important for the well-being and survival of young children, is not all that matters. Children from relatively high-income households are better-taken care of and might be at a lesser risk of diseases regardless of their health insurance status. Second, because insurance affects health outcomes by reducing the price of healthcare, households from different income groups will have different sensitivities to the lower price. This affects the take-up rate of the NHIS differently for the various groups. The NHIS in Nigeria is effective in improving the health outcomes of children. Findings from the study show a large effect on the life expectancy of children from low-income households. Policies strengthening the take-up of the NHIS should be encouraged across all sectors and socio-economic groups in the economy.

## Appendices

APPENDIX A

OCCUPATIONS IN THE PUBLIC SECTOR AND OTHER SECTORS

The public sector in the analysis comprises of the following occupations

1. Civil servants, council workers and directors

2. Civil defense and/or security service workers

3. Community Liaison officers

4. Diplomats

5. Forest/ conservation workers

6. Government administrators

7. Government executive officials

8. Judges, magistrates, jurists, judiciary workers, and legal assistants

9. Elected officials, politicians and legislative officials

10. Mail distributors and related workers

11. Military Personnel, fire fighters, policemen and detectives

12. National Electric Power Authority (NEPA) workers

13. National Youth Service Corps (NYSC) members

14. National Drug Law Enforcement Agency (NDLEA) workers

15. Nigerian Union of Petroleum and Natural gas (NUPENG) workers/ trade unionist

16. Primary/public health workers

17. Protective service workers

18. Social welfare workers

Other sectors comprise of the following occupations

1. Not working

2. New workers seeking employment

3. Merchants and shopkeepers, wholesale and retail traders

4. Salesmen, shop assistants and street vendors

APPENDIX B

THE DID (Difference in Difference) ESTIMATOR FOR THE NHIS IMPLEMENTATION IN THE PUBLIC SECTOR

The expected outcome for children in the public sector ($Public_{ist} = 1$) before the NHIS implementation and expansion ($Postimp_{it} = PostExp_{it} = 0$) is ($\beta _{0} + \beta _{3} + \beta _{6}X_{ist}+\delta ^{!}NDHSYear_{ist}+\varphi ^{!}Month_{ist}+\gamma ^{!}State_{ist}$). The expected outcome for children in the public sector after the implementation ($Postimp_{ist}=1$) after the implementation $Postimp_{ist}=1$) but before the expansion ($PostExp_{it}=0)$ is $(\beta _{0}+\beta _{1}+\beta _{3}+\beta_{4}+\beta _{6}X_{it}+\delta ^{!}NDHSYear_{it}+\varphi ^{!}Month_{ist}+\gamma ^{!}State_{ist})$ The difference in outcomes for public sector children is $(\beta _{1}+\beta _{4}).$. This is the sum of the time and treatment effect. Also, the expected outcome for children in other sectors $({Public_{ist}=0})$ is before the NHIS implementation and expansion $(Postimp_{ist}=PostExp_{ist}=0)$ is $(\beta _{0}+\beta _{6}X_{ist}+\delta ^{!}NDHSY_{ist}+\varphi ^{!}Month_{ist}\gamma ^{!}State_{ist}).$. The expected outcome for children in other sectors $(PostExp^{ist}=0)$ is $(\beta _{0}+\beta _{1}+\beta _{6}X_{ist}+\delta ^{!}NDHSYear_{ist}+\varphi ^{!}Month_{ist}+\gamma ^{!}State_{ist})$. The difference in outcomes for children in other sectors is $(\beta _{1})$. This is the pure time effect. The DID estimator for the NHIS implementation is therefore $(\beta _{1}+\beta _{4})-(\beta _{1})=\beta_{4}.$
